# Multilevel Barriers to Perianal Condyloma Care Among Men Who Have Sex With Men in Northeast China

**DOI:** 10.1001/jamanetworkopen.2025.45768

**Published:** 2025-11-26

**Authors:** Fangxiao Zhang, Tingting Lu, Xiaoyun Hu, Yingqi Zhao, Yalun Li

**Affiliations:** 1 Department of Infectious Diseases II, First Affiliated Hospital of China Medical University, Shenyang, Liaoning, China; 2 Department of Anorectal Surgery, First Affiliated Hospital of China Medical University, Shenyang, Liaoning, China; 3 Department of Pharmacology, School of Pharmacy, China Medical University, Shenyang, Liaoning, China

## Abstract

**Importance:**

Men who have sex with men (MSM) with perianal condyloma face compounded health care challenges within structural inequities. Current research poorly characterizes their medical experiences, including barriers to care, psychosocial stressors, and policy-driven exclusion. Human papillomavirus (HPV)–related perianal condyloma in MSM is associated with high recurrence, diagnostic delays, and limited preventive access, underscoring the need to address systemic disparities.

**Objective:**

To explore health care experiences of MSM with perianal condyloma, identify multilevel care barriers, and characterize structural oppression affecting clinical outcomes.

**Design, Setting, and Participants:**

This qualitative study used semistructured interviews and qualitative analysis. Twenty MSM with confirmed perianal condyloma acuminatum were purposively sampled via clinician referrals. Inclusion criteria included age of 18 years or older and the capacity to provide informed consent. The study was conducted at a tertiary hospital (First Affiliated Hospital of China Medical University, Northeast China). Data were collected from January 1 to March 31, 2025, and analyzed from March 31 to June 30, 2025.

**Main Outcomes and Measures:**

Thematic analysis and grounded theory were used to identify barriers across 4 levels (individual, clinical, societal, and policy) and reflect how multiple barriers contribute to structural oppression.

**Results:**

Twenty MSM (mean [SD] age, 23.95 [4.36] years) participated in the study. Of the 20 MSM, 10 (50%) were students, 17 (85%) had a bachelor’s degree or higher, 13 (65%) identified as gay, and 9 (45%) engaged in exclusive anal-receptive intercourse. The mean (SD) monthly income was ¥4725 (¥2573). Regarding health care visits, 8 (40%) visited for active disease, 5 (25%) used private health care, and 1 (5%) had HIV coinfection. Participants faced synergistic barriers: (1) gaps in HPV knowledge, reliance on unverified online information, and risky sexual behaviors (individual level); (2) fragmented care pathways, privacy violations, and postoperative trauma (clinical level); (3) stigma-driven identity concealment, social isolation, and relationship strain (societal level); and (4) exclusion from HPV vaccination, lack of male-adapted screening tools, and inadequate sexual health education (policy level). Together, these barriers reflect how clinical neglect and structural oppression emerge and how their intersection perpetuates health care disparities among MSM with perianal condyloma.

**Conclusions and Relevance:**

In this qualitative study of MSM with perianal condyloma, the findings suggest that privacy-protected care, standardized protocols, inclusive prevention strategies, and practitioner cultural competency training are required. These findings also emphasize the urgent need for equity-focused sexual health policies to address the needs of marginalized populations.

## Key Points

**Question:**

What are the health care dilemmas and structural exclusions faced by men who have sex with men (MSM) with perianal condyloma during medical visits?

**Findings:**

This qualitative study of 20 MSM with perianal condyloma identified multilevel barriers (individual, clinical, societal, and policy), including human papillomavirus knowledge gaps, fragmented care, social stigma, and policy exclusion among MSM, and presents key observations that align with the concept of structural oppression influencing care outcomes.

**Meaning:**

This study suggests that multilevel interventions (privacy protection, standardized care, inclusive prevention, and practitioner training) are needed to advance health equity for sexual minority populations.

## Teaser

This qualitative study of men who have sex with men who have perianal condyloma seeks to identify gaps in culturally responsive care and propose evidence-based interventions to address the unique health challenges of this population.

## Introduction

Condyloma, caused by human papillomavirus (HPV) infection, is clinically characterized by high recurrence risk and substantial psychosocial burden.^1^ Epidemiologic data show that most condyloma cases are attributed to HPV-6/11, with mucocutaneous transmission elevating infection risks in sexually active populations.^2,3^ Notably, men who have sex with men (MSM) exhibit a significantly higher incidence of perianal HPV infection, a disparity attributed to anatomical characteristics and anal intercourse practices.^4^ The unique infection susceptibility and diagnostic complexity of perianal condyloma in MSM require population-specific care pathways.

Of particular concern, perianal condyloma in MSM is associated with unique clinical challenges: high recurrence rates, diagnostic delays due to concealed lesion location, and limited access to standardized care.^5^ Standard care for condyloma includes surgical excision, topical therapies, and surveillance protocols.^6^ However, these interventions often fail to address psychosocial impacts, such as stigma-driven care avoidance, which exacerbates disease progression. HPV-related malignant neoplasms share a pathogenetic origin with condyloma but represent an advanced disease stage.^7,8,9,10^

Sexual and gender minority (SGM) populations, especially MSM, face multifaceted health care barriers that pose critical public health challenges.^11,12^ Social stigma undermines health-seeking behaviors, causing diagnostic delays and compromising public health efficacy.^13,14^ Beyond stigma, systemic inequities persist. The absence of competency training for SGM-specific care in health professions education has perpetuated gaps in culturally adaptive health care delivery.^15,16,17,18^ Public health policies further entrench inequities, with MSM facing discriminatory insurance criteria (eg, denial of life or critical illness coverage) and exclusionary coverage that violate health equity principles.^19,20^ Unequal distribution of medical resources has created substantial gaps in targeted care, as seen in suboptimal HPV vaccination coverage among MSM despite its proven safety and efficacy.^21,22^ Policy formulations often neglect gender diversity, mismatching preventive measures with population needs, whereas critical gaps in sexual health education and community outreach further hinder health communication.^23,24^ Institutional discrimination, resource inequities, and health literacy deficits collectively create “health care deserts” that impede equitable access for MSM.^25,26^

This qualitative study aims to bridge this gap by exploring the medical experiences of MSM with perianal condyloma. We examine health care barriers, psychosocial challenges, and unmet preventive needs through patient narratives. By integrating these perspectives, we seek to identify gaps in culturally responsive care and propose evidence-based interventions to address the unique health challenges of this population.

## Methods

### Design, Setting, and Sample

This qualitative study used semistructured interviews to explore perianal condyloma care experiences among MSM, with data analyzed via thematic analysis and grounded theory. The study was conducted at the First Affiliated Hospital of China Medical University (a Northeast China tertiary hospital) and involved 20 MSM with a confirmed perianal condyloma diagnosis. Participants were purposively sampled through clinician referrals, with the following inclusion criteria: aged 18 years or older, confirmed diagnosis of perianal condyloma, and capacity to provide informed consent. Relapses were defined as the reappearance of genital warts requiring clinical treatment and removal. Data collection took place from January 1 to March 31, 2025, continuing until data saturation was achieved at the 20th participant. This sample demonstrated diversity in education, occupation, and income. The China Medical University institutional review board approved this study, and written informed consent was provided by all participants.

### Data Collection

Semistructured interviews (50-60 minutes) were audio-recorded with informed consent in private settings. Recordings were professionally transcribed verbatim within 72 hours, yielding 539 inductively coded analyzable units. Participants received health education packages and hygiene products (valued at <¥100 [<US $14]), compliant with institutional ethics noncoercive reimbursement guidelines.^27^ A tripartite security protocol was implemented: (1) deidentification with alphanumeric codes after collection, (2) physical documents in double-locked cabinets, and (3) digital files on password-protected servers. Access was restricted to investigators involved in this study.

### Data Analysis

The software MAXQDA, version 24 (VERBI Software GmbH) was used to manage and code the qualitative data. Initially, 2 authors (F.Z. and Y.L) independently performed inductive open-axial-selective coding on the data, integrating Braun and Clarke’s^28^ 6-phase thematic analysis process and Strauss and Corbin’s^29^ iterative coding principles. Coding logic was iteratively refined through cross-checks between coders. Any inconsistencies in coding assignments or theme definitions were resolved by a joint review of the original records. After the initial independent coding, 2 other authors (T.L. and X.H.) reviewed the coded data and preliminary thematic framework to verify the consistency of coding logic and topic clustering. This study followed the Consolidated Criteria for Reporting Qualitative Research (COREQ) reporting guideline to ensure comprehensive and transparent reporting of all critical aspects of the qualitative research process.^30,31^ Data saturation was determined when no new themes emerged from the analysis of interview data (until the 20th participant), and all collected interviews were fully analyzed to confirm that no additional meaningful elements were overlooked.

## Results

### Baseline Characteristics

Among 20 MSM with perianal condyloma (mean [SD] age, 23.95 [4.36] years) included in the study (Table 1), 10 were students (50%) 17 (85%) had a bachelor’s degree or higher. Thirteen (65%) identified as gay, 7 (35%) identified as bisexual, and 14 (70%) concealed their orientation. Nine (45%) exclusively practiced anal-receptive intercourse, and 11 (55%) combined anal-receptive and penile-insertive intercourse. Monthly income ranged from ¥1000 to ¥10 000 (mean [SD], ¥4725 [¥2573]). Clinical visits were for active disease (8 [40%]) or follow-up (12 [60%]), with a mean (SD) of 1.95 (2.26) relapses. Private health care use was reported by 5 (25%), and 1 (5%) had HIV coinfection. Based on the coded results (Table 2), participants encountered multifaceted barriers across 4 levels, with each set of barriers playing a role in shaping the oppression affecting their health care experience. (Figure).

**Table 1.** Participant Characteristics

Characteristic	Participants, No. (%)
Age, mean (SD), y	23.95 (4.36)
Occupation	
Student	10 (50)
Nonstudent	10 (50)
Educational attainment	
Bachelor’s degree or higher	17 (85)
Less than bachelor’s degree	3 (15)
Sexual orientation	
Gay	13 (65)
Bisexual	7 (35)
Sexual behavior patterns	
Anal-receptive intercourse	9 (45)
Anal-receptive and penile-insertive intercourse	11 (55)
Disclosure of sexual orientation	
Yes	6 (30)
No	14 (70)
Individual monthly income, mean (SD) [range], ¥	4725.00 (2572.50) [1000-10 000]
Reason for visit	
Active disease visit	8 (40)
Follow-up examination	12 (60)
No. of relapses, mean (SD) [range]	1.95 (2.26) [0-8]
Experience with private health care facilities	
Yes	5 (25)
No	15 (75)
Coinfected with HIV	
Yes	1 (5)
No	19 (95)

**Table 2.** Selective-Axial-Open Coding

Theme	Conceptual definition
**Individual level**	
Limitations in disease cognition	
Lack of disease knowledge	Insufficient knowledge of HPV or perianal condyloma
Risk perception bias	Selective neglect of personal perianal condyloma risk factors (eg, MSM identity, multiple sexual partners)
Misconceptions about prevention	Misunderstanding condom efficacy; confusion between HIV and HPV infection prevention
High-risk sexual behavior	
Cognitive deficit–induced risk negligence	Unawareness of transmission risks due to knowledge gaps, leading to weakening of safe sex practices
Risk disregard due to optimism bias	Conscious risk recognition with optimism-driven safety neglect
Partner trust–induced risk disregard	Voluntary safety reduction in trusted sexual partnerships
Disconnected health management	
Misconceptions about perianal diseases	Self-misdiagnosis of hemorrhoids and other anal diseases and medication use leading to delayed diagnosis and treatment
Prevention priority bias	Imbalanced focus on HIV or other STI monitoring vs HPV prevention
Absence of self-testing tools	Lack of male-specific HPV test kits hindering self-monitoring
Sexual partner notification failure	Public health tracking failures due to lost contact with partners or refusal to screen
Complex network information	
Internet information dependence	Self-help learning that relies on social media and online platforms to obtain fragmented knowledge and information
Information overload and noise	Misleading medical advice from online misinformation
Experience-driven care selection	Preference for hospitals or physicians based on online patient community experiences
**Societal level**	
Internalized stigma	
Delayed medical treatment	Health care delays due to sexual minority identity conflicts
Psychological maladjustment	Respondents exhibit clinically significant anxiety and stress
Transmission anxiety	Moral distress in disease transmission scenarios
Intimacy disruption	Relationship breakdowns due to blame for transmission; challenges in sustaining sexual needs
External social exclusion	
Perceived stigma	Fear of social discrimination and misconceptions about promiscuity
Disease concealment	Hiding the disease from family and friends, feeling lonely during the treatment
Privacy concerns in treatment	Fear of diagnostic label exposure or data leaks in medical settings
Clinical stress	
Fear of disease progression	Anxiety over recurrence, prolonged treatment, and uncertain outcomes
Hypervigilance	Excessive self-examination and hypochondriac behaviors
Sexual identity adaptation	Worrying about or even quitting their sexual behavior patterns, and feeling conflicted about their sexual identity
Financial toxicity	Economic burden from recurrent treatment costs
Support strategies	
Support from people around	Encouragement and care from friends, family and partners
Physician-patient trust	Reduced anxiety through privacy-protected care and physician professionalism
Adaptive disease reconciliation	Gradually come to terms with recurring illnesses and cultivate positive cognitive shifts throughout treatment
Therapeutic disclosure	Be honest with physicians about sexual orientation and MSM sexual behaviors to receive effective medical treatment
**Medical level**	
Poor medical resource accessibility	
Deficiency in specialized medical resources	Insufficient specialists and uneven regional distribution
Ambiguous health care navigation	Ambiguous departmental division of labor and inadequate public disclosure of doctors’ qualifications for perianal condyloma treatment
Distrust in telemedicine	Concerns about online care accuracy and privacy
Time and travel burden	Recurrent care demands and time/geographical burdens compromise follow-up adherence
Systemic health care governance anomie	
Guideline discordance	Non–evidence-based treatment regimens (eg, nonguideline drugs) and the insufficient standardization of diagnosis and treatment operations
Decline in patient trust	The erosion of patient-physician trust is exacerbated by physicians’ deceptive clinical behaviors (eg, deliberate exaggeration of diagnoses) and systemic deficiencies in professional competence
Medical consumption trap	Systematic inducement of unnecessary medical services coupled with coercive overpriced service practices in private clinics
Dignity violations	Privacy exposure during treatment process, encountering discriminatory attitudes from medical staff
Posttreatment challenges	
Physical trauma	Postoperative complications and pain
Impaired quality of life	Insomnia and attentional deficits resulting in impaired work performance and diminished quality of life
Self-care barriers	Self-isolation or living alone, postoperative self-care practice barriers
**Policy level**	
Prevention system gaps	
Lack of vaccine education	Limited public awareness of HPV vaccine benefits and access
Vaccine policy restrictions	Policy barriers to male HPV vaccination
Vaccine skepticism	Misconceptions about vaccine efficacy
Shortcomings in sexual health education	
Weak perception of disease	The absence of collective awareness in the MSM community stems from a collective unconsciousness of HPV risks and a breakdown in health information exchange
Absence of health education outreach	Lack of authoritative channels for learning and addressing questions, alongside deficiencies in school-based sexual health education
Barriers to dissemination of health information	
Negative learning attitude	Low participation intention caused by cognitive bias in the value of sexual health education
Communication form requires innovation	Digital platforms, such as short videos and public accounts, are more popular in health communication
Communication among patients	Overreliance on internal communication within patient communities
Institutional marginalization	
Health rights marginalization	Sexual minorities feel that the development of the medical system does not focus enough on their medical needs
MSM-friendly health care requirements	Calling for the improvement of the medical consultation environment to make it more inclusive for sexual minorities
Systemic health care avoidance	Concerns over career repercussions from HPV medical records lead some to abandon insurance, avoid formal care, and even turn to hidden medical treatment

Abbreviations: HPV, human papillomavirus; MSM, men who have sex with men.

**Figure.**
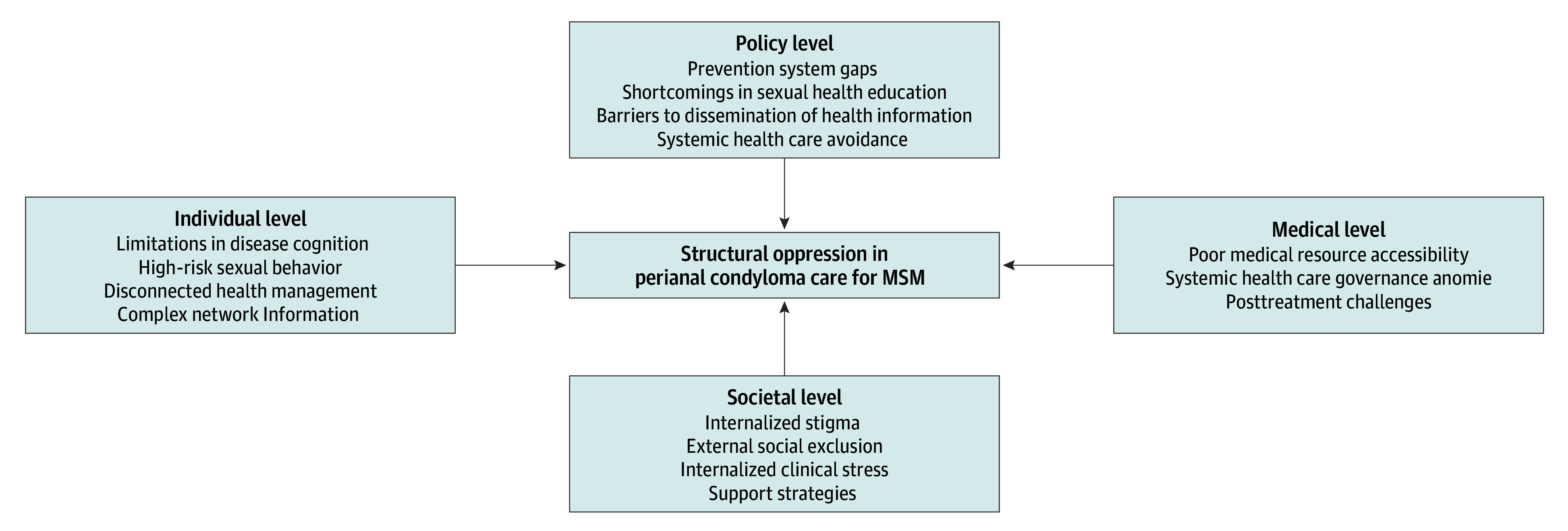
Multilevel Barriers Contributing to Structural Oppression in Perianal Condyloma Care for Men Who Have Sex With Men (MSM)

### Individual Level

The interviews revealed that people do not know perianal condyloma is a common phenomenon, from the HPV pathogens, transmission routes, and the lack of comprehensive knowledge of susceptible population. One participant stated, “I always thought that HPV could only be infected by women.” (Participant 4) Another participant noted, “Who would have thought homosexual sexual behavior could spread HPV?” (Participant 20) These statements illustrate how the lack of knowledge about transmission led to underestimated infection risks. This cognitive deficit then manifested in excessive reliance on condoms as a sole protective measure. Another participant admitted, “[I] only realized after getting HPV that condoms are not foolproof.” (Patient 11)

Misunderstandings about HPV latency and recurrence mechanisms contributed to insufficient attention to sexual health. Moreover, relational trust in sexual partners further weakened protective measures. One participant said, “Most guys focus on diseases like HIV and syphilis, and don’t pay much attention to HPV.” (Participant 15) Another stated, “I trusted my partner so much that I never checked his health. Otherwise, I would’ve taken precautions.” (Participant 14) The lack of early symptom recognition compounded the issue, as seen in a participant’s self-misdiagnosis: “At first, I thought it was hemorrhoids...used topical ointment myself.” (Participant 6)

Under the dual pressures of knowledge gaps and identity-related stigma, online platforms became the primary information source. However, unregulated online content posed new risks because it not only failed to bridge knowledge deficits but also introduced misleading treatment guidance. One participant said, “Many treatment methods on the internet...don’t know which one is suitable.” (Participant 1) Another participant stated, “Some medical promoters would approach me online posing as patients and recommend some medicines, all of which were products from unreliable small workshops.” (Participant 19) These findings revealed that among MSM with perianal condyloma lack of knowledge about the disease leads to high-risk sexual behaviors and insufficient attention to sexual health, whereas MSM are more prone to complex online information, which further aggravates their confusion about HPV knowledge, thus perpetuating the cycle.

### Societal Level

Social disapproval of MSM identities compelled patients to hide their condition from family and friends, leading to social isolation. One participant admitted, “I hide my meds in snack bags so family won’t find them.” (Participant 14) Meanwhile, the risk of privacy breach in medical settings triggered medical avoidance. A participant described their experience with the examination room: “Packed with med students...nearly walked out then and there.” (Participant 20) The conflict between sexual orientation privacy and health care needs undermined patients’ access to support in both personal relationships and health care systems.

The sexual transmission nature of perianal condyloma introduced another layer of complexity to interpersonal relationships. Ethical conflicts threatened the stability of intimate relationships, and fear of disease led to sexual withdrawal. One participant avoided sexual activity: “Although I have sexual needs, I’m more afraid of getting sick.” (Participant 18) Meanwhile, the high recurrence rate of perianal condyloma and concealed location warts exacerbated anxiety driven by relapse concerns. Another participant spent nights obsessively checking for recurrence: “Every night, I’d search online, take photos, observe for signs of recurrence, and stay up until exhausted.” (Participant 8)

However, the analysis also incorporates countervailing forces. Although family and friend support provided some relief, the lack of deep emotional resonance often rendered it insufficient. As one participant noted, “Friends can only offer verbal comfort, which is of little help.” (Participant 17) In contrast, the establishment of trust with physicians emerged as a critical mediating factor. Another participant said, “Unlike the previous doctors, the experience and attitude of my current attending physician make me feel at ease here for treatment.” (Participant 3)

External social exclusion and clinical stress, along with internalized stigma, combine to form multisource stressors for MSM patients. These stressors, from both nonmedical social pressures and medical-related concerns, intertwine to create a difficult situation. Although support strategies help to some extent, patients find it hard to counterbalance the harms caused by such widespread and intertwined pressures.

### Medical Level

#### Diagnostic Stage

Specialized HPV screening and standardized care were concentrated in tertiary hospitals, creating resource disparities. One participant stated, “Why can’t I find a place that can be treated seriously?” (Participant 5) This regional imbalance, coupled with unclear departmental responsibilities, created difficulties in seeking medical advice. Another participant expressed confusion: “Some say go to anorectal, others dermatology. The dermatologist couldn’t check inside the anal canal—where should I go?” (Participant 16) The high recurrence rate of perianal condyloma necessitated frequent medical visits, increasing time and economic costs. Although telemedicine offered convenience, diagnostic uncertainty and privacy concerns limited its use. One participant distrusted telemedicine: “Online doctor asked for photos, but the advice was confusing; I couldn’t trust it.” (Participant 8)

#### Treatment Stage

The absence of medical standardization allowed for variable treatments. The heavy reliance on individual physicians’ experience for lesion removal increased the risk of incomplete treatment, as seen in one participant’s recurrence: “Many warts recurred. Obviously doctor did not treat them thoroughly last time.” (Participant 19) Another participant faced forced consumption: “[The doctor] asked me to take particularly expensive medicines, otherwise wouldn’t treat me.” (Participant 2)) Information asymmetry further enabled unethical commercial behaviors, with some physicians exploiting patients’ situations. Privacy violations were also evident. One participant recalled, “The doctor blurted my diagnosis in a crowded waiting room.” (Participant 19)

#### Rehabilitation Stage

Postoperative pain aggravated the fear of recurrence and subsequent diagnosis and treatment. One participant said, “The pain was killing me, all I could do was lie in bed...skipped eating because I was too scared to use the toilet.” (Participant 13) Disease anxiety led to psychosocial impairment, thereby seriously affecting the quality of life. One participant felt frustrated: “My appetite has become poor and I always lack energy. I used to be the top student in the college, but my grades dropped a lot in the last exam.” (Participant 12)

From seeking care to receiving treatment, each stage of the entire medical process presents distinct obstacles and difficulties. These cumulative barriers collectively undermine MSM patients’ access to timely, high-quality, and respectful medical care.

### Policy Level

The exclusion of male patients from HPV vaccine policies created a structural deficit in preventive care. One participant mentioned, “Some wealthy people will go abroad to get vaccinated...the vaccine is not allowed for men here.” (Participant 2) Another misunderstood: “I think the vaccine is useless for men.” (Participant 11) Some respondents were not even aware of the existence of the HPV vaccine, exposing failures in science communication about HPV prevention.

The long-term avoidance of comprehensive sexual health education in schools and the general public created knowledge gaps. MSM relied on informal channels due to insufficient official guidance. As one participant described, “Chinese sex education has been weak since childhood...short videos are quicker and more straightforward.” (Participant 16) Another participant was criticized for sharing his experience: “Netizens bashed me for saying I was showing off. But with no authoritative guidance, what else are we supposed to do?” (Participant 17) Patients explicitly called for medical institutions to launch new media accounts to bridge this gap.

Targeted services for MSM were almost nonexistent, with privacy violations rampant. One participant demanded, “Hospitals need privacy-protected wards.” (Participant 1) Digital health care compounded this: patients feared electronic records would disclose sexual orientation, forcing them to choose between support and privacy. Another patient forwent medical insurance to avoid career risks: “Medical records might expose my sexuality, affecting my job.” (Participant 9) MSM face comprehensive policy-driven marginalization within the health care system, which extends beyond inadequate hospital infrastructure to encompass underdevelopment in multiple areas outside clinical settings, including prevention initiatives, sexual health education, insurance access, and health information dissemination.

## Discussion

Structural oppression theory has been applied in medical education and health equity studies.^32,33,34^ As a prevalent sexually transmitted disease among MSM, perianal condyloma exemplifies the long-standing structural health disparities faced by SGM populations in sexual health care.^35,36^ Qualitative analysis revealed that individual risk behaviors, psychosocial oppression, fragmented medical resources, and policy exclusion interact synergistically. This interaction exacerbates HPV disease progression and accumulates health risks and challenges. These findings contribute to health equity theory by first highlighting the contextual specificity of perianal condyloma care disparities in MSM, second identifying intervention nodes through the interplay of multilevel barriers, and third offering a transferable analytical approach to other stigmatized health conditions. These contributions provide intervention direction to address health inequities among SGM groups.

Among MSM, anal intercourse is a characteristic behavior that facilitates HPV exposure to the susceptible mucosal tissues of the anal canal and perianal area, thereby increasing the risk of perianal condyloma.^35^ This study confirms widespread HPV knowledge gaps in this population, undermining prevention awareness.^37,38^ Notably, even patients with basic safety awareness show reduced vigilance regarding perianal condyloma, likely due to HPV’s long incubation period, concealed symptoms, and perceived lower risk compared with high-profile infections such as HIV.

Sexual health education has gained global attention but remains inadequate and overly sensitive for SGM populations in countries such as China.^39,40,41^ Although new media is the primary source of knowledge for MSM, it has significant limitations. The lack of authoritative policy-supported platforms and regulatory gaps directly compromises information authenticity, a finding consistent with prior research.^42,43^ Patients with perianal condyloma relying on experience-sharing create homogeneous echo chambers, where repeated circulation of nonsystematic knowledge confirms that knowledge deficits primarily formed by systemic information barriers and are not solely attributable to individual negligence. This dynamic inherently reflects the structural nature of health care oppression, rooted in systemic marginalization of MSM.

Specialist shortages and their concentration in major hospitals create regional resource disparities, a manifestation of structural flaws in the health care system that weakens perianal condyloma management in primary care.^44,45^ Even in tertiary hospitals, collaboration deficits and unclear referral guidelines force patients to navigate multiple clinics.^46^ Anal HPV infection, mainly caused by low-risk HPV-6/11, receives limited clinical attention.^47^ Some clinicians are reluctant to serve MSM due to epidemiologic biases, whereas patients face a difficult choice between privacy protection and accessing health care. This mutual avoidance widens the service gap: despite increasing societal inclusivity, both medical system failures and individual attitudes exacerbate access challenges.

MSM patients’ psychological burden stems from 3 core sources: (1) sexual identity stigma fuels fears of having sexual behavior misunderstood, (2) the lack of humanistic care of medical staff worsens medical experiences, and (3) the risks of perianal condyloma recurrence and treatment trauma induce persistent anxiety.^48^ More profoundly, failures in the support system have emerged. Familial and friend support offers minimal emotional relief. The absence of medical psychological care forces patients to rely on closed patient networks. The nonstandardization and commercialization in medicine erode patient-physician trust.^4,6^ Clinicians’ attitude biases toward SGM can be traced back to the insufficient humanistic training in medical education.^49,50^ Studies^51,52^ confirm that SGM populations have elevated physical and psychological morbidity. This stems not only from disease pathology but also from multilevel management failures. The convergence of social moral pressure, deficits in medical humanism, and gaps in policy oversight has turned treatable psychosocial issues into unmanaged crises.

Global HPV vaccine coverage among males remains low, with MSM excluded from national immunization programs.^53,54^ This not only exacerbates their high infection risk but also undermines preventive public health measures, forcing health care systems to address needs inefficiently under resource constraints. Notably, during this study, China approved the first quadrivalent HPV vaccine (Merck Sharp & Dohme) for males aged 9 to 26 years (January 8, 2025), which will bring some improvements in the future. Anal canal warts are difficult to detect early, and this is compounded by the lack of male-specific HPV self-test kits.^55,56^ Commercial self-testing tools are still largely designed for women, severely restricting safe and effective self-screening among MSM. Systematic marginalization and technical barriers in prevention methods have disconnected prevention intentions from practice, effectively excluding MSM from health protection systems.

Multipronged interventions are critical to address the structural health care oppression faced by MSM with perianal condyloma. In response to systemic privacy risks faced by MSM, privacy protection should be prioritized through the establishment of confidential medical environments and strict enforcement of information security protocols, thereby eliminating core barriers to care. Perianal condyloma clinical pathways should be optimized by building multidisciplinary networks with clearly defined interdepartmental roles to tackle inefficiencies in the medical system. A dual-track strategy should be implemented to overcome prevention gaps and misinformation: male HPV vaccination programs are advocated for early intervention, and new media can be used as a sexual health platform where MSM can receive accurate information.^57^ Lasting change to rectify biases rooted in medical education requires integrating sexual minority sensitivity training into health care professionals’ continuing education, thereby reducing implicit biases.^58^ These recommendations offer a targeted program to address sexual health inequities and have the potential to be adapted to other marginalized health domains as an inclusive policy paradigm.

### Limitations

This qualitative study exploring health care barriers among MSM with perianal condyloma has inherent limitations. Qualitative design limits generalizability; quantitative studies are needed to quantify barriers. Recruitment from a single hospital in Northeast China may limit geographic generalizability. The sample skewed toward younger students may not adequately represent older MSM or those with diverse occupational backgrounds. Additionally, because our participants were limited to those already seeking care, there would be a potential selection bias. However, this limitation partly reflects a practical contradiction: MSM who do not seek care are inherently difficult to identify clinically. Future studies should prioritize age, occupational, and socioeconomic diversity to generate more representative insights.

## Conclusions

This study found that MSM with perianal condyloma confront multilevel barriers across individual, clinical, societal, and policy dimensions. Critical gaps in HPV knowledge and reliance on unreliable health information undermine prevention efforts and timely care-seeking. Fragmented clinical pathways, nonstandardized treatments, and privacy violations in medical systems further impede effective perianal condyloma management. Social stigma exacerbates psychological distress, forcing concealment of sexual identity and health status. Structural exclusion from HPV vaccination programs and the absence of male-specific self-test tools deepens preventable infection risks.

Disrupting this oppression requires integrated interventions focused on structural oppression faced by MSM: prioritizing privacy-protected care, standardizing clinical protocols, implementing inclusive prevention strategies, and enhancing cultural competency training for medical staff. These findings emphasize the urgent need for equity-focused sexual health policies to address the needs of marginalized populations.

## Notes

Supplement.
Data Sharing Statement
