# Supplementary material for: Multilevel Barriers to Perianal Condyloma Care Among Men Who Have Sex With Men in Northeast China
Source: JAMA Netw Open. 2025 Nov 26;8(11):e2545768. doi: 10.1001/jamanetworkopen.2025.45768 (PMC12658667; doi:10.1001/jamanetworkopen.2025.45768)
Supplement: Supplement. — Data Sharing Statement [file jamanetwopen-e2545768-s001.pdf]

Data Sharing Statement

Zhang. Multilevel Barriers to Perianal Condyloma Care Among Men Who Have Sex With Men in Northeast China. *JAMA Netw Open*. Published November 26, 2025.
doi:10.1001/jamanetworkopen.2025.45768

Data
Data available: No
